# Investigation on the Biaxial Flexural Strength of Universal Shade Resin-Based Composites

**DOI:** 10.3390/polym16131853

**Published:** 2024-06-28

**Authors:** Keiko Sakuma, Taku Horie, Takafumi Kishimoto, Mayumi Maesako, Shigetaka Tomoda, Morioki Fujitani, Akimasa Tsujimoto

**Affiliations:** 1 Department of Operative Dentistry, School of Dentistry, Aichi Gakuin University, Nagoya 464-8651, Japan; virgo@dpc.agu.ac.jp (K.S.); taka-ki@dpc.agu.ac.jp (T.K.); maesako@dpc.agu.ac.jp (M.M.); fusafusa@dpc.agu.ac.jp (S.T.); morioki@dpc.agu.ac.jp (M.F.); aki-tj@dpc.agu.ac.jp (A.T.); 2 BIOMAT—Biomaterials Research Group & UZ Leuven (University Hospitals Leuven), Dentistry, KU Leuven (University of Leuven) Department of Oral Health Sciences, 3000 Leuven, Belgium; 3 Department of Operative Dentistry, University of Iowa College of Dentistry, Iowa City, IA 52242, USA; 4 Department of General Dentistry, Creighton University School of Dentistry, Omaha, NE 68102, USA

**Keywords:** biaxial flexural strength, universal shade, resin-based composite

## Abstract

The biaxial flexural strength of universal shade and conventional dental resin-based composites before and after alkaline degradation was investigated. Disk samples were prepared from these resin-based composites, and some of the specimens were immersed in 0.1 M NaOH solution to create deteriorated samples. The biaxial flexural strength of the samples before and after the alkaline degradation test was measured and statistically tested. The fracture surfaces after the biaxial flexural test were observed using a scanning electron microscope. The results showed that the biaxial flexural strength of the paste-type universal shade resin-based composite before alkaline degradation was significantly (19%) higher than that of the conventional type, but no difference was observed between the materials after alkaline degradation. On the other hand, the biaxial flexural strength of the flowable universal shade resin-based composites was significantly (around 35%) lower than that of the conventional composite, with or without degradation. Although, for paste-type materials, the biaxial flexural strength of universal shade resin-based composites was higher than that of conventional resin-based composites before alkaline degradation, after degradation the two materials showed similar values. For flowable materials, the biaxial flexural strength of universal shade resin-based composites was lower than that of conventional resin-based composites regardless of the presence or absence of degradation processes. These results suggest that some caution should be used when deciding whether to use flowable universal shade resin-based composite to fill a cavity.

## 1. Introduction

Resin-based composites are a class of material that embody the important philosophy of minimally invasive dentistry [1]. This philosophy was first proposed by FDI in 2002 and has since been refined and developed in multiple dimensions, including types of adhesive, techniques, and restorative materials [2]. Resin-based composites are part of the development of materials to support minimally invasive dentistry and have been developed by applying the results of a wide range of laboratory research over many studies to clinical practice [3]. Research focusing on improving bonding performance between the resin-based composite and the tooth substrate has led to significant progress in bond durability through improvements in adhesive systems [4], which has made it possible to achieve restorative treatments that minimize loss of sound tooth structure. In addition to the rapid spread of the concept of minimal intervention dentistry, increasing aesthetic demands in restorative dentistry also stimulated the increase in the use of resin-based composites tremendously, as these composite materials are much closer in appearance to natural tooth tissue than amalgam or metal restorations, while being significantly cheaper than indirect ceramic restorations.

However, one of the important issues that is still a challenge to overcome in resin-based composites is the quality of the color match between the resin-based composite and the tooth substrate [5]. Natural teeth consist of layers of different materials with different optical properties — enamel is translucent, while dentin is more opaque — and it is difficult to mimic this with a single substance [6]. In addition, various factors, such as tooth hue, type, cavity shape and patient age, need to be consider for precise matching of color with the surrounding tooth structure [7,8]. Earlier work has managed to improve the color match by layering resin-based composites pigmented in different color shades to match dentin and enamel, and in this way it is possible to thoroughly match the color of the surrounding tooth structure [9]. However, the complexity of the layering technique and the need to choose appropriate colors for each layer means that this approach requires a high level of clinical skill and experience to achieve high esthetic results. Also, using resin-based composites of different colors with a layering technique does not always yield the expected results. Moreover, the standardization needed to achieve the intended results with the layering technique is not found among manufacturers [10]. Color formation problems associated with the layering technique, potential variations depending on layering thickness and order, and the optical differences among manufactures’ products are all challenging for clinicians.

The color of resin-based composites is determined by various parameters, such as hue, chroma, brightness and translucency, which are general components of color [6,11]. The color-related characteristics of materials constitute a phenomenon known as color compatibility. Matching between restorative material and tooth structure is defined as color interaction associated with layering and color transition, occurring at different stages such as during production or placement, and subsequent to it, including color stability, as well as the emission of a color similar to composite materials by surrounding tooth structure [12]. Clinically, when observed together, color differences between tooth structure and aesthetic restorative materials are perceived to be less pronounced than they are when viewed separately. When materials adopt the color of the surrounding tooth structures, the overall aesthetic appearance of restorations improves [13]. It is assumed that this effect arises from color shifting due to the reflection of light and color from the surrounding tooth structures in resin-based composite restorations. This phenomenon is commonly referred to as the “chameleon effect” [14]. This effect occurs due to the mutual color reflections between the tooth structure and the resin-based composite restoration [15]. The extent of it is dependent on the light transmission properties of the resin-based composite, enamel margin configuration, and the mutual orientation of light with enamel prisms [16]. Fundamentally, light transmitted through the resin-based composite partially reflects back from the base and sidewalls of the cavity. The dispersed light reflected from the sidewall of the cavity contributes to blurring the boundaries of the restoration, while the light reflected straight from the cavity base subtly adjusts the perceived color of the restoration.

Recently introduced universal shade resin-based composites are designed to seamlessly blend with existing tooth structure and render color matching more predictable [17]. These materials feature enhanced translucency that can reflect the surrounding tooth color within the composite. As a result, this optical phenomenon enables monochromatic resin-based composite to effectively match the natural tooth structure in various clinical situations, thereby facilitating better color adaptation [5]. Based on this innovative concept, manufacturers claim that with universal shade resin-based composite resins, there is no need for color selection in direct restorative treatment procedures. Therefore, the frequency of the clinical use of universal shade resin-based composites in restorative dentistry has increased due to their simple usage. Although these resin-based composites have the same basic composition of matrix resin and filler as conventional resin-based composites, they generate the color of the restorative material in a fundamentally different way. Rather than employing inherently colored pigments distributed throughout the material, they use the mechanism of structural coloration, which is said to assimilate the color of the tooth structure [18]. These universal shade resin-based composites are characterized by the production of filler particles of a consistent shape and size using the sol–gel method, resulting in a uniform dispersion of supra-nano spherical particle fillers, with a particle size of 260 nm, along with organic-inorganic hybrid fillers [19,20]. The consistent shape and size of these fillers means that their uniform dispersal interacts with incident light to create color within the material through the diffraction of that light. In addition, thanks to not containing pigments of their own, they can also take on the color of the surrounding tooth structure, which is visible through the largely colorless material. This means, in theory, they can provide a color match more easily than conventional pigmented resin-based composite.

Although most of the previous studies on universal shade resin-based composites have focused on color compatibility and color change between the restoration and tooth structure [21,22], there have been few studies on the mechanical properties of these resin-based composites. The two most common methods used to determine the flexural properties of dental restorative materials are the three-point bending test and the biaxial test methods. The mechanical properties of resin-based composites have often been evaluated using the three-point bending test, because this method for resin-based composites was technically standardized for evaluating the flexural properties of resin-based composites by International Standardized Organization (ISO) 4049 [23,24]. Currently, ISO 4049 is recognized as a screening testing method for evaluating the flexural properties of resin-based composites and requires a beam specimen of 25 × 2 × 2 mm for the three-point bending test. Because of the large specimen dimensions, multiple overlapping curing is necessary to light cure the resin-based composites, which may lead to inhomogeneity of the samples. Within the overlapping irradiation zones, more radicals are generated from the reaction between the activator and the photo initiator, which results in higher degree of polymerization as compared with the adjacent regions. In addition, it is technically difficult to prepare flaw-free samples at this length. Any voids or irregularities present in the materials would result in uneven stress distribution within the specimen, which will influence the observed strength.

On the other hand, the biaxial flexural test method has been commonly used to evaluate the flexural properties of fragile dental ceramic materials [25,26]. Over the last almost half century, an increasing number of applications of this test method with dental restorative materials have been reported. Since the 1980s, piston-on-three-ball and ball-on-ring biaxial flexure measurements have been performed on dental ceramic materials, light-cured resin-based composites, and glass-ionomer cements (GIC) [27]. Recently, the biaxial flexural test method has been also applied to study the flexural strength of dental zirconia [25].

However, no study has evaluated the mechanical properties of universal shade resin-based composites using the biaxial flexural test, which is mainly used to measure the flexural properties of ceramic restorative materials and is technically specified by the ISO [28]. The biaxial flexural test was standardized for dental ceramic materials through the ISO in 2015. The flexural stress generated in a restoration tends to be concentrated in the center of the specimen and the test is conducted in water, to evaluable the flexural properties in a way that simulates the oral environment as far as possible. Ban et al. [29] used this testing method to evaluate zinc phosphate, polycarboxylate, glass ionomer, silicate, and zinc oxide-eugenol cements, and thus the effectiveness and broad applicability of this testing method has been widely demonstrated.

This study presents the investigation of the biaxial flexural strength of paste- or flowable-type universal shade and conventional resin-based composites before and after alkaline degradation.

## 2. Materials and Methods

### 2.1. Materials

Universal shade and conventional resin-based composites of the paste and flowable types from the same manufacturer (Tokuyama Dental, Tokyo, Japan) were used in this study. Omnichroma (OC, Tokuyama Dental) was used as the universal shade resin-based composite of the paste type, and Estelite Σ Quick (ES, Tokuyama Dental) was used as the conventional comparison resin-based composite. Omnichroma Flow (OCF, Tokuyama Dental) was used as the flowable-type universal shade resin-based composite, and Estelite Universal Flow (ESF, Tokuyama Dental) was used as the conventional comparison resin-based composite. Different manufacturers use fundamentally different ingredients and techniques in their resin-based composites, meaning that if the materials come from multiple manufacturers, it is not possible to determine the influence of the difference between paste and flowable type, and between conventional and universal shade resin-based composites. These materials were chosen because materials of similar composition were available in all four types. The shade of the paste and flowable conventional resin-based composites was A3, and the consistency type of ESF was Medium. Again, differences in shade can affect composition and thus material properties, so it was important to be consistent. The resin-based composites used are listed in Table 1.

### 2.2. Sample Preparation

**Figure 1:**
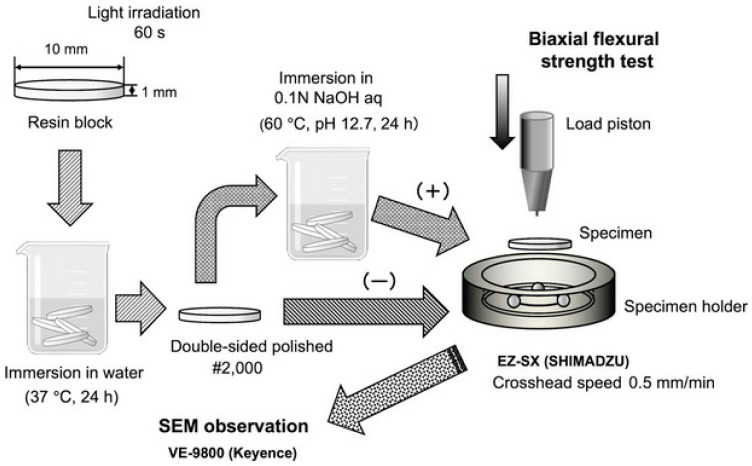
Experimental procedure.

The experimental procedure is shown in Figure 1. Teflon molds (inner diameter 10 mm, height 1 mm) were filled with each resin-based composite, and then the molds were pressed with a glass slide through a polyethylene film and were irradiated with a light curing unit (Valo Curing Light, Ultradent Products, South Jordan, UT, USA) for 60 s to prepare resin block samples. The resin block samples were stored in distilled water at 37 °C for 24 h in a constant temperature chamber, to simulate intra-oral conditions. Both sides of the samples were then polished in an automatic rotary polisher (Ecomet 3000, Buehler, Lake Bluff, IL, USA) using water-resistant silicon carbide paper up to #2000 under water injection to prepare disk-shaped specimens. In addition, some of the samples were immersed in 0.1 M NaOH solution (60 °C, pH 12.7) for 24 h and used as deteriorated specimens. These samples were used as biaxial flexural test samples, and the number of samples was 10 for each condition.

### 2.3. Biaxial Flexural Test

The samples were mounted on a biaxial flexural test fixture and were fractured using the EZ Test compact tabletop testing machine (EZ-SX, Shimadzu Corp., Kyoto, Japan) at a crosshead speed of 0.5 mm/min. The fracture stress was calculated from the measured fracture load values using the equations defined in ISO 6872: 2015, and Poisson’s ratio was set to 0.24 [10]. The maximum fracture stress calculated was used as the biaxial flexural strength for each group.

### 2.4. Scanning Electron Microscopy (SEM) Observation

Ultrastructural observations were conducted on the polished surfaces of the resin-based composites using SEM. The samples were ultrasonically cleaned for 5 min, and the surface of each sample was sputtered with 10 nm-thick gold (MSP-1S, Vacuum Device, Ibaraki, Japan) before SEM observation. SEM observations were conducted using a scanning electron microscope (VE-9800, Keyence, Osaka, Japan) with an accelerating voltage of 5 kV.

### 2.5. Statistical Analysis

The obtained biaxial flexural strengths were analyzed using statistical software (IBM SPSS, Chicago, IL, USA). After checking the normality of the data (Shapiro–Wilk test), data were independently evaluated for the paste type (OC and EQ) and the flowable type (OCF and EQF) using the resin-based composite (material) and the presence or absence of alkaline degradation (condition) as the influencing factors, and a two-way analysis of variance and multiple comparison test at a significance level of α = 0.05 were conducted. Statistical tests were performed (α = 0.05).

## 3. Results and Discussion

### 3.1. Biaxial Flexural Strength

The results of the two-way ANOVA for the paste-type resin-based composites (OC and ES) and the mean (standard deviation) biaxial flexural strength for each group are shown in Table 2 and Table 3, respectively. Although the biaxial flexural strength of the paste-type resin-based composites was affected by the material (*p* = 0.006) and the presence of alkaline degradation (*p* < 0.001), there was no interaction between these factors (*p* = 0.083). The biaxial flexural strength of both OC and ES was significantly lower under alkaline-degraded conditions than under non-alkaline-degraded conditions. Th biaxial flexural strength of OC was significantly higher than that of ES under the non-alkaline condition, but there was no significant difference between OC and ES under the alkaline-degraded condition.

The results of the two-way ANOVA for the flowable resin-based composites (OCF and ESF) and the mean (standard deviation) biaxial flexural strength for each group are shown in Table 4 and Table 5, respectively. Although the biaxial flexural strength of flowable resin-based composites was affected by the material (*p* < 0.001) and the presence of alkaline degradation (*p* < 0.001), there was no interaction between these factors (*p* = 0.611). The biaxial flexural strength of both OCF and ESF was significantly lower under alkaline-degraded conditions than under non-alkaline-degraded conditions. The biaxial flexural strength of OCF was significantly lower than that of ESF, regardless of whether alkaline degradation was applied or not.

### 3.2. SEM Observations

**Figure 2:**
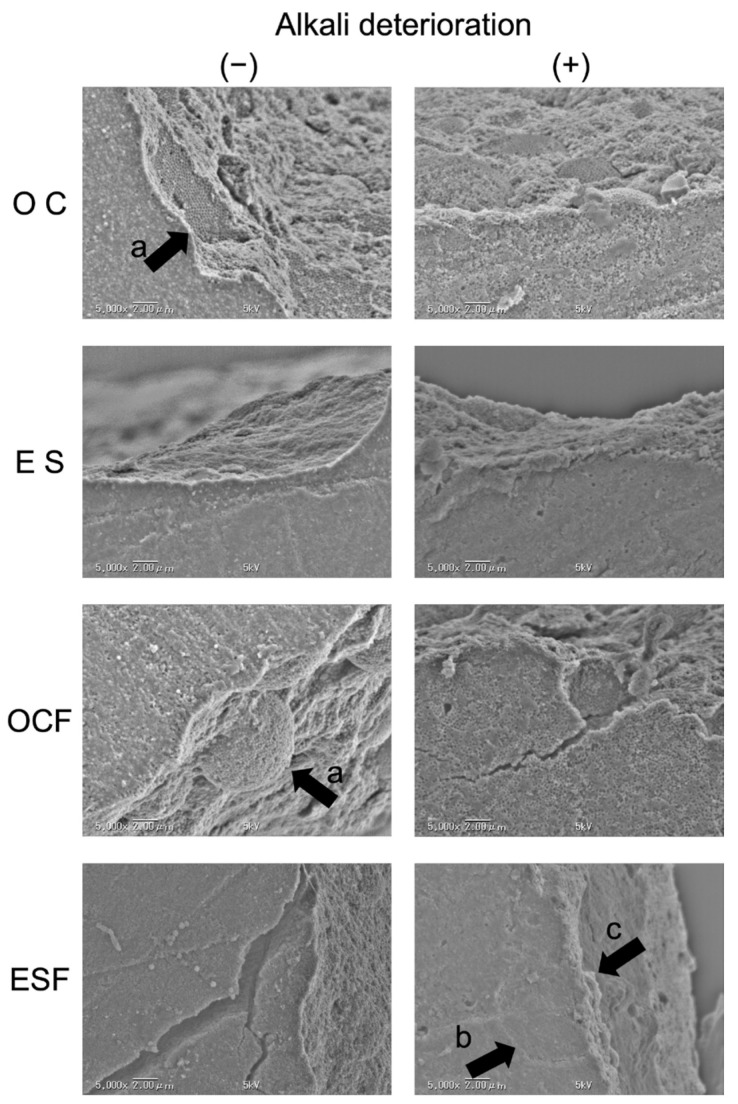
Representative SEM observations of samples after biaxial flexural strength tests. (a) Exfoliation of spherical particle fillers and organic-inorganic hybrid fillers was observed. (b) Delamination along the organic-inorganic hybrid filler was observed. (c) Fracture penetrating the interior of the organic-inorganic hybrid filler was observed.

Representative SEM observations of fracture surfaces after the biaxial flexural test are shown in Figure 2. In the paste-type resin-based composites, OC showed a characteristic fracture surface, where spherical particle fillers and organic-inorganic hybrid fillers composed of spherical particle fillers detached from the matrix resin. After alkaline degradation, this was even more prominent. In ES, some loss of spherical particle fillers was also observed at the fracture surface, but it was not prominent even under alkaline-degraded conditions.

In the flowable resin-based composites, OCF showed a detachment of the spherical particle fillers and the organic-inorganic hybrid filler composed of spherical particle fillers from the matrix resin for OC. Evidence was observed suggesting that the fracture had started at the boundary between the filler and the resin matrix, in particular, the boundary between the organic-inorganic hybrid filler and the resin matrix. In ESF, as in ES, the spherical particle filler was observed to be detached from the resin matrix, although the detachment was not obvious. In the alkaline-degraded sample, fractures following the borders of the organic-inorganic hybrid filler particles were observed, but so were fractures passing through those particles.

### 3.3. Discussion

The flexural test, in which a bending moment is applied to a material, is one of the evaluation methods for the mechanical properties of resin-based composites, and the three-point bending test has been frequently used in their evaluation [23,30]. In this study, the biaxial flexural test, which is often used to evaluate the flexural properties of ceramics [31], was used to add novel insights to the evaluation of the flexural properties of universal shade resin-based composites.

Generally, the flexural strength of resin-based composites needs to fulfill the minimum requirement specified in ISO 4049 (flexural strength > 50 MPa) and the flexural strength values obtained in the biaxial flexural test would be expected to be higher than those obtained by three point bending test, as the sample has smaller dimensions. This difference in measured flexural strength was also previously reported with a study conducted on ceramic restorative materials [32]. It has been reported that the discrepancy can be attributed to the difference between the two test configurations in the effective area or volume of the material subjected to maximum tensile stress [32]. The span-to-thickness ratios used in the three-point bending and piston-on-three-ball biaxial test are 10:1 and 5.7:1, respectively. Therefore, the biaxial test, which has a much lower effective area under maximum stress directly under the loading ball, may be expected to result in a higher strength value. On the other hand, a larger stress distribution occurs in the three-point bending test, which has a larger effective area between the support spans. Thus, the material may be more vulnerable to failure due to localized imperfections in the three-point bending test. In this study, the flexural strength of paste type resin-based composites with and without the application of the degradation process was found to be 103.7-156.2 MPa and that of flowable type resin-based composites with and without the application of the degradation process was found to be 135.6-240.8 MPa. Thus, even though we would expect to see higher values than those measured with the three-point bending test, and thus should apply a higher standard that specified by the ISO for the three-point test, the present values would seem to meet the minimal requirements for clinical use, as even the lowest results exceed a value of 50 MPa by at least a factor of two.

Four common loading methods for the biaxial flexural test have been identified as standard support and loading combinations: ball-on-three-ball, piston-on-three-ball, ring-on-ring and ball-on-three-ball A previous study reported that, based on study results and finite element analysis, the ball-on-ring and ball-on-three-ball loading methods were capable of accurately determining the flexural strength of dental brittle materials, while uncertain fracture stresses were found in the other methods, leading to inaccurate results [32]. However, the piston-on-three ball loading method is describing in ISO 6872 [28]. Thus, in order to maintain consistency with the ISO 6872 method, the piston-on-ball loading method was used in this study. In addition to the loading method, the thickness of the sample is one of the most important factors in the biaxial test, as it affects the degree of deflection and distribution of the stress. In ISO 6872 for dental ceramic materials, a sample thickness of 3.0 mm (2.1 ± 1.1 mm) is recommended. However, in this study, the biaxial strength of resin-based composites, which are more flexible than the ceramic materials, was evaluated. Therefore, in this study, the minimal thickness (1.0 mm) specified by ISO 6872 was used to determine the biaxial flexural strength while minimizing the deflections of the material. 

In paste-type resin-based composites, this study found that the flexural strength of OC (universal shade) was significantly higher than that of ES (conventional). Although the biaxial flexural strength values of both resin-based composites decreased significantly after alkaline degradation, there was no significant difference between the two types of material after degradation. The reason for the difference in biaxial flexural strength for the resin-based composites before alkaline degradation is thought to be due to differences in the composition of the resin-based composites. Maesako et al. [33,34] have claimed that the transmission of incident light is important for universal shade resin-based composites that enable structural coloration, and that the interface (filler-resin matrix coupling agent) between the filler (dispersive phase) and the resin matrix (organic phase) must be designed to be strong so as not to affect the reflection and interference of incident light. To achieve this strong bonding, it is thought to be important to clean the filler and to perform a reliable silane treatment corresponding to nano-sized fillers. Although the bonding condition between the filler and resin matrix in OC is therefore expected to be good, it is possible that damage may occur under conditions of extreme degradation. The results of the study showed that the filler and resin matrix were well bonded in OC.

The alkaline degradation test used in this experiment is a method that forcibly promotes the hydrolysis of the silane coupling, causing the filler to detach. Although this testing method creates an environment that cannot occur in the oral cavity, it allows filler bonds to be broken quickly, permitting observations of the starting point of degradation [35,36]. In the SEM observations made in this experiment, the bond between the filler and resin matrix was significantly degraded in the alkaline-degraded resin-based composite, suggesting that the biaxial flexural strength after degradation may be the same, despite differences in the composition of the two materials.

In the biaxial flexural strength results for flowable-type resin-based composites, contrary to the paste type, the value for the universal shade OCF was significantly lower than that of the conventional ESF, regardless of the presence or absence of alkaline degradation. The biaxial flexural strength of universal shade flowable resin-based composite was significantly lower than that of the conventional flowable resin-based composite in the results of this experiment. This suggests that although the shape, particle size, and amount of filler in the universal shade flowable resin-based composite, which enable structural coloration, have been optimized to achieve structural coloration as in the paste type, it might not be easy to ensure adequate mechanical properties. Therefore, in the future development of universal shade resin-based composites, it is desirable to develop materials with excellent mechanical properties equivalent to those of recent flowable resin-based composites, as well as with excellent color compatibility.

Previous studies have found a wide range of values for the maximum bite force in the mouth, 650.7–762.5 N in men and 553.0–652.5 N in women [37,38]. Restorations may not be exposed to the maximum force, but previous work has found that the fatigue limit is typically half of the maximum force that a material can withstand [4]. Converted into Newtons, the forces at fracture in this experiment ranged from 84.4 N to 168.5 N. These are clearly lower than the forces that might be expected during mastication, and so these results indicate that these materials should not be used to restore surfaces that will be directly exposed to occlusal forces.

## 4. Conclusions

- For paste-type materials, the biaxial flexural strength of universal shade resin-based composites was higher than that of conventional resin-based composites before alkaline degradation, but after degradation, the two materials showed similar values.
- For flowable materials, the biaxial flexural strength of universal shade resin-based composites was lower than that of conventional resin-based composites.
- Flowable universal shade resin composites should be used with caution in dental restorations.

## Figures and Tables

**Table 1:** Resin-based composites used in this study.

Brand Name	Omnichroma	Omnichroma Flow (Medium)	Estelite Σ Quick	Estelite Universal Flow
Code	OC	OCF	ES	ESF
Manufacturer	Tokuyama	Tokuyama	Tokuyama	Tokuyama
	Dental	Dental	Dental	Dental
Lot No.	573	48,032	219,089	94,040
Filler size	φ260 nm	φ260 nm	φ200 nm	φ200 nm
Filler contents	79 wt% (68 vol/%)	71 wt% (57 vol/%)	82 wt% (71 vol/%)	71 wt% (57 vol/%)
Matrix resin	UDMA	UDMA	Bis-GMA	Bis-GMA UDMA Bis-MPEPP

Abbreviations: Bis-GMA, bisphenol A diglycidyl methacrylate; Bis-MPEPP, 2,2-Bis(4-methacryloxypolyethoxyphenyl)propane; UDMA, urethane dimethacrylate.

**Table 2:** Results of two-way ANOVA in paste-type resin-based composites.

Source	SS	df	Mean Squares	F-Ratio	*p*-Value
Material	140,487.357	3	46,829.119	190.593	0.006
Condition	20,302.466	1	20,302.466	82.63	<0.001
Material × Condition	1470.167	3	490.056	1.99	0.083

**Table 3:** Biaxial flexural strengths in paste-type resin-based composites.

Condition	Material	
	OC	ES
Alkaline-deterioration (−)	156.2 (28.6) ^Aa^	131.2 (9.9) ^Ab^
Alkaline-deterioration (+)	109.7 (5.2) ^Ba^	103.7 (7.9) ^Ba^

Units are MPa. Values in parentheses are standard deviations. The same upper-case letter in columns indicates no significant difference (*p* > 0.05). The same lower-case letter in rows indicates no significant difference (*p* > 0.05).

**Table 4:** Results of two-way ANOVA in flowable resin-based composites.

Source	SS	df	Mean Squares	F-Ratio	*p*-Value
Material	61,679.818	1	61,679.818	290.642	<0.001
Condition	7139.851	1	7139.818	33.644	<0.001
Material × Condition	55.909	1	55.909	0.263	0.611

**Table 5:** Biaxial flexural strengths in flowable resin-based composites.

Condition	Material	
	OCF	ESF
Alkaline-deterioration (−)	160.0 (10.6) ^Aa^	240.8 (8.7) ^Ab^
Alkaline-deterioration (+)	135.6 (12.4) ^Ba^	211.72 (20.5) ^Bb^

Units are MPa. Values in parentheses are standard deviations. The same upper-case letter in columns indicates no significant difference (*p* > 0.05). The same lower-case letter in rows indicates no significant difference (*p* > 0.05).

## Data Availability

Data are contained within the article.
